# Differential roles of RIG-I like receptors in SARS-CoV-2 infection

**DOI:** 10.1186/s40779-021-00340-5

**Published:** 2021-09-07

**Authors:** Duo-Meng Yang, Ting-Ting Geng, Andrew G. Harrison, Peng-Hua Wang

**Affiliations:** Department of Immunology, School of Medicine, University of Connecticut Health Center, Farmington, CT 06030 USA

**Keywords:** SARS-CoV-2, Pathogen pattern recognition receptor, Melanoma differentiation-associated protein 5, Retinoic acid-inducible gene I

## Abstract

Retinoic acid-inducible gene I (RIG-I) and melanoma differentiation-associated protein 5 (MDA5) sense viral RNA and activate antiviral immune responses. Herein we investigate their functions in human epithelial cells, the primary and initial target of severe acute respiratory syndrome coronavirus 2 (SARS-CoV-2). A deficiency in MDA5, RIG-I or mitochondrial antiviral signaling protein (MAVS) enhanced viral replication. The expression of the type I/III interferon (IFN) during infection was impaired in *MDA5*^−/−^ and *MAVS*^−/−^, but not in *RIG-I*^−/−^, when compared to wild type (WT) cells. The mRNA level of full-length angiotensin-converting enzyme 2 (ACE2), the cellular entry receptor for SARS-CoV-2, was ~ 2.5-fold higher in *RIG-I*^−/−^ than WT cells. These data demonstrate MDA5 as the predominant SARS-CoV-2 sensor, IFN-independent induction of ACE2 and anti-SARS-CoV-2 role of RIG-I in epithelial cells.

**Supplementary Information:**

The online version contains supplementary material available at 10.1186/s40779-021-00340-5.

## Dear Editor,

Severe acute respiratory syndrome coronavirus 2 (SARS-CoV-2) is an enveloped, positive sense single-stranded RNA virus that has caused the greatest global public health crisis in the twenty-first century. Its pathogenesis remains largely unknown—highlighting a critical need for new research in this area. The cytoplasmic retinoic acid-inducible gene I (RIG-I) like receptors (RLRs) are major pattern recognition receptors (PRRs) for RNA viruses. Once engaged by viral RNA, RLRs bind mitochondrial antiviral signaling protein (MAVS), which ignites a signaling cascade, leading to transcription of immune genes [1]. Because of their importance to initiation of antiviral immune responses, these pathways are thus common targets of immune evasion by many viruses including SARS-CoV-2 [2].

We investigated the role of RLRs in controlling SARS-CoV-2 infection and mounting immune responses in a human lung epithelial cell line Calu-3. We generated individual knockouts using CRISPR-Cas9, and validated them by immunoblotting (Additional file 1: Fig. S1a). To prove that these gene functions are precisely silenced, we infected mutant cells with vesicular stomatitis virus (VSV, specifically activates RIG-I-MAVS) with a green fluorescence protein (GFP) integrated into its genome. As expected, *RIG-I*^−/−^ or *MAVS*^−/−^ cells presented a higher VSV-GFP load than wild type (WT) cells, while *MDA5*^−/−^ cells had a similar viral load as WT cells (Additional file 1: Fig. S1b). We then compared SARS-CoV-2 load and interferon (IFN) in these cells. The intracellular viral RNA loads were significantly higher in all knockout cells than WT cells at 24 and 72 h post infection (p.i.) (Additional file 2: Fig. S2a). Consistently, the extracellular viral titers produced by all knockout cells were also higher than those by WT cells (Additional file 2: Fig. S2b). We confirmed these observations in another human lung epithelial cell line A549 (Additional file 2: Fig. S2c), though which is significantly less permissive to SARS-CoV-2. Although primarily sensing DNA viruses, the cyclic GMP-AMP synthase (cGAS)-stimulator-of-interferon-genes (STING) signaling pathway also restricts many RNA virus infection [3]. We noted a slight increase in SARS-CoV-2 load in *STING*^−/−^ cells (Additional file 2: Fig. S2d, e), suggesting that STING signaling is largely dispensable for control of SARS-CoV-2.

We next examined antiviral immune responses. The *IFNB1* (type I IFN) and *IL29* (type III IFN) mRNA levels were continuously upregulated during the course of infection in WT cells; while this induction was impaired in *MDA5*^−/−^ and *MAVS*^−/−^ cells, so was one of interferon-stimulated genes (ISG15) (Additional file 3: Fig. S3a). The concentrations of IFN-λ and C-X-C motif chemokine ligand 10 (CXCL10) proteins in the cell culture supernatants from *MDA5*^−/−^ and *MAVS*^−/−^ were much lower than WT cells (Additional file 3: Fig. S3b). However, type I/III IFN and ISG15 expression was higher in *RIG-I*^−/−^ than WT cells (Additional file 3: Fig. S3a), suggesting that RIG-I interferes with SARS-CoV-2 replication independently of IFNs. We next examined if RLR signaling regulates expression of angiotensin-converting enzyme 2 (ACE2), the predominant cellular entry receptor for SARS-CoV-2, thus influences viral replication. In the airway epithelium, in addition to full-length ACE2 (805 amino acids), a short isoform (459 amino acid) without the 17 aa of the signal peptide and 339 aa of the N-terminal peptidase domain is expressed. The short form, but not full-length, is inducible by type I/III IFNs. However, the short isoform fails to bind the SARS-CoV-2 spike protein, thus likely has no role in viral entry. We first quantitated full-length ACE2 using our own primers (targeting Exon 4 and 5) by quantitative RT-PCR. Of note, the mRNA level of full-length ACE2 was induced by over twofold in WT at 24 and 72 h when compared to 1 h p.i. It was also induced in *MDA5*^−/−^ and *MAVS*^−/−^ as normally as in WT cells (Additional file 3: Fig. S3c), though these knockout cells were deficient in type I/III IFN expression (Additional file 3: Fig. S3a, b), suggesting that SARS-CoV-2 infection induces ACE2 expression in an IFN-independent manner. Intriguingly, it was ~ 2.5 fold higher in *RIG-I*^−/−^ than WT cells throughout the course of infection (Additional file 3: Fig. S3c), suggesting that RIG-I might suppress full-length ACE2 transcription. We confirmed these results using a published primer pair for full-length ACE2 only (Additional file 3: Fig. S3d). We next assessed the expression of the short isoform with two unique pairs of primers according to recent studies, which designated it MIRb and dACE2 respectively. The short isoform was upregulated in WT cells by > 3.5 times at 72 h, when compared to 1 h p.i., however, it was not induced at all in *RIG-I*^−/−^ cells (Additional file 3: Fig. S3d).

Understanding the major PRR pathways in the respiratory tract epithelial cells is physiologically meaningful as these cells are the first line of host defense. The RLR signaling is functional in all tissues and cell types, in contrast to viral RNA-sensing TLR3/7 that are primarily limited to immune cells. Our results demonstrate that MDA5 is the predominant RLR for SARS-CoV-2, consistent with two recent studies [4, 5]. However, in neither MDA5 nor MAVS knockout cells, induction of IFNs was completely abolished, suggesting that other PRRs may collectively play a role. RIG-I deletion had no negative impact on IFN responses, but still enhanced viral replication, suggesting that RIG-I plays a MAVS-IFN-independent antiviral role. However, the role of RIG-I in SARS-CoV-2 infection is inconsistent. Yin et al. [4] demonstrated that RIG-I was dispensable for the control of SARS-CoV-2 replication, while both our and Yamada’s [5] data suggested otherwise. Mechanistically, RIG-I likely binds the 3’ untranslated region of the SARS-CoV-2 RNA genome via its helicase domains and prevents viral RNA replication independently of IFNs [5]. In addition to the above-mentioned mechanisms, our results suggest that RIG-I could restrain full-length ACE2 expression, consequently SARS-CoV-2 cellular entry. To our surprise, induction of the short isoform of ACE2 expression by SARS-CoV-2 seems dependent on RIG-I. Although the mechanism underlying the contrasting role of RIG-I in full-length/short ACE2 transcription remains unknown, notably, their transcription is indeed regulated differently. Comprehensive future work is necessary to elucidate this.

We want to point out that our findings are limited to human lung epithelial cell lines, and other PRRs such as viral RNA-sensing TLR3/7 may be important SARS-CoV-2 sensors in other cell types. Nonetheless, given the essential role of MDA5 in initiation of antiviral immune responses in the airway epithelium, the MDA5 agonists could thus be potentially therapeutic against early SARS-CoV-2 infection.

## Supplementary Information

**Additional file 1: Fig. S1**. Functional validation of gene knockouts by CRISPR-Cas9. **a** The immunoblots show gene knockout efficiency in Calu-3 cells. β-actin is a housekeeping gene and serves as a protein loading control. **b** Fluorescent microscopic images of VSV-GFP at several time points post infection (p.i.). Magnification: 100 × . The results are representative two reproducible independent experiments. GFP green fluorescence protein, MAVS mitochondrial antiviral signaling protein, MDA5 melanoma differentiation-associated protein 5, RIG-I retinoic acid-inducible gene I, VSV vesicular stomatitis virus.

**Additional file 2: Fig. S2**. An important role of the MDA5-MAVS axis in control of SARS-CoV-2 infection. **a** Quantitative RT-PCR analyses of SARS-CoV-2 RNA loads in Calu-3 cells infected with SARS-CoV-2 at a multiplicity of infection (MOI) of 0.5. **b** The extracellular viral titers in the cell culture supernatants of Calu-3 cells. **c** Quantitative RT-PCR analyses of SARS-CoV-2 RNA loads in A549 cells infected with SARS-CoV-2 at a MOI of 0.5. **d** The immunoblots show STING knockout efficiency in Calu-3 cells. β-actin is a housekeeping gene and serves as a protein loading control. **e** Quantitative RT-PCR analyses of SARS-CoV-2 RNA loads in Calu-3 cells infected with SARS-CoV-2 at a MOI of 0.5. All the data are presented as mean ± SEM and statistical significances are analyzed by one-way ANOVA. The results are representative two reproducible independent experiments, *n* = 3–4 in each group. Compared with WT, *P < 0.05; **P < 0.01; ***P < 0.001. MAVS mitochondrial antiviral signaling protein, MDA5 melanoma differentiation-associated protein 5, PFU plaque forming unit, RIG-I retinoic acid-inducible gene I.

**Additional file 3: Fig. S3**. An essential role of the MDA5-MAVS axis in induction of type I/III IFNs by SARS-CoV-2. **a** Quantitative RT-PCR analyses of immune gene transcripts. **b** Quantification of IFN-λ and CXCL10 proteins by ELISA, in Calu-3 cells infected with SARS-CoV-2 at a multiplicity of infection (MOI) of 0.5. **c** Quantitative RT-PCR analyses of full-length ACE2 mRNA. **d** The short isoform of ACE2. MIRb ACE2 and dACE2 are different designations for the same short isoform from two recent publications. All the data are presented as mean ± SEM and statistical significances are analyzed by one-way ANOVA (**a** and **b**), and non-parametric Mann–Whitney *U* test (**d**). The results are representative two reproducible independent experiments, *n* = 3 for each group. Compared with WT, *P < 0.05; **P < 0.01; ***P < 0.001; ****P < 0.0001. Compared with 1 h, ^#^*P* < 0.05; ^##^*P* < 0.01. ACE2 angiotensin-converting enzyme 2, CXCL10 C-X-C motif chemokine ligand 10, IFNB1 type I IFN, IFN interferon, IL29 type III IFN; ISG15 interferon-stimulated gene 15, MAVS mitochondrial antiviral signaling protein, MDA5 melanoma differentiation-associated protein 5, RIG-I retinoic acid-inducible gene I.

## Data Availability

All relevant data and materials are within this paper and its additional files.

## Abbreviations

ACE2: Angiotensin-converting enzyme 2
cGAS: Cyclic GMP-AMP synthase
CXCL10: C-X-C motif chemokine ligand 10
GFP: Green fluorescence protein
IFN: Interferon
ISG15: Interferon-stimulated gene 15
MAVS: Mitochondrial antiviral signaling protein
MDA5: Melanoma differentiation-associated protein 5
MOI: Multiplicity of infection
PFU: Plaque forming unit
p.i.: Post infection
PRR: Pathogen pattern recognition receptor
RIG-I: Retinoic acid-inducible gene I
RLR: Retinoic acid-inducible gene I like receptor
SARS-CoV-2: Severe acute respiratory syndrome coronavirus 2
STING: Stimulator-of-interferon-genes
TLR: Toll-like receptor
VSV: Vesicular stomatitis virus
WT: Wild type
