# Supplementary figures and images for: Differential roles of RIG-I like receptors in SARS-CoV-2 infection
Source: Mil Med Res. 2021 Sep 7;8:49. doi: 10.1186/s40779-021-00340-5 (PMC8421188; doi:10.1186/s40779-021-00340-5)

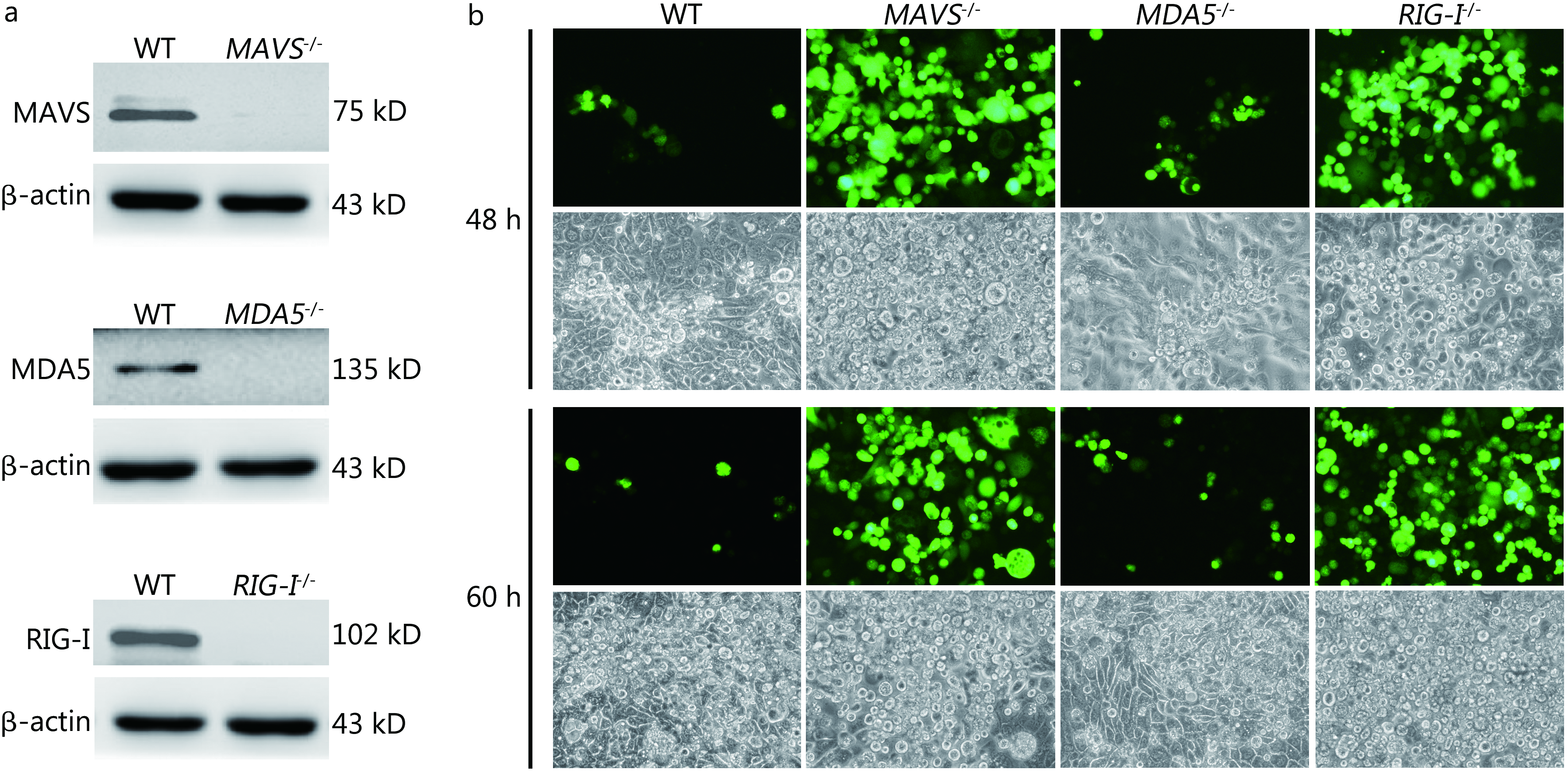

Supplement: Supplementary file 1 — Additional file 1: Fig. S1. Functional validation of gene knockouts by CRISPR-Cas9. a The immunoblots show gene knockout efficiency in Calu-3 cells. β-actin is a housekeeping gene and serves as a protein loading control. b Fluorescent microscopic images of VSV-GFP at several time points post infection (p.i.). Magnification: 100 × . The results are representative two reproducible independent experiments. GFP green fluorescence protein, MAVS mitochondrial antiviral signaling protein, MDA5 melanoma differentiation-associated protein 5, RIG-I retinoic acid-inducible gene I, VSV vesicular stomatitis virus. [file 40779_2021_340_MOESM1_ESM.tif]

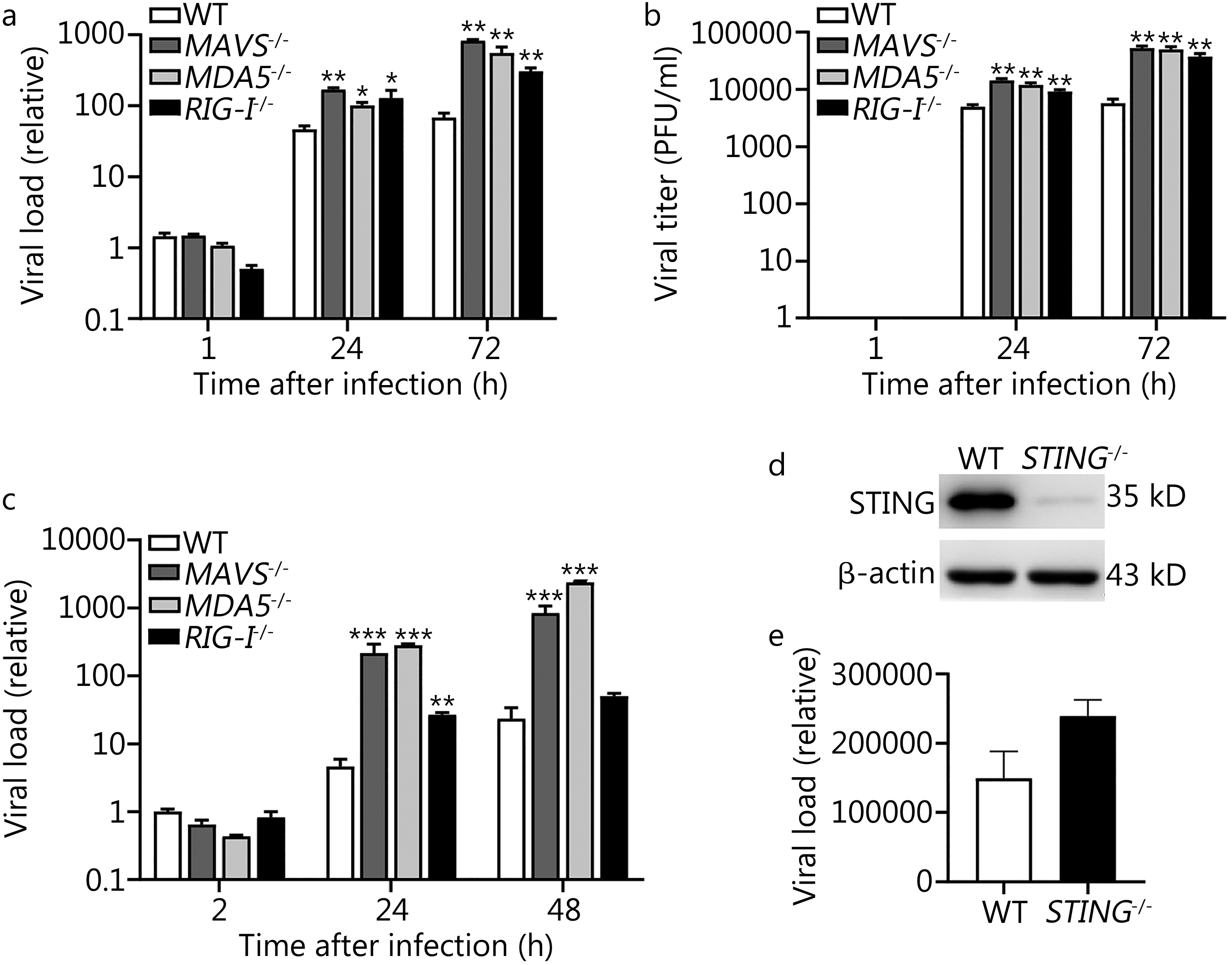

Supplement: Supplementary file 2 — Additional file 2: Fig. S2. An important role of the MDA5-MAVS axis in control of SARS-CoV-2 infection. a Quantitative RT-PCR analyses of SARS-CoV-2 RNA loads in Calu-3 cells infected with SARS-CoV-2 at a multiplicity of infection (MOI) of 0.5. b The extracellular viral titers in the cell culture supernatants of Calu-3 cells. c Quantitative RT-PCR analyses of SARS-CoV-2 RNA loads in A549 cells infected with SARS-CoV-2 at a MOI of 0.5. d The immunoblots show STING knockout efficiency in Calu-3 cells. β-actin is a housekeeping gene and serves as a protein loading control. e Quantitative RT-PCR analyses of SARS-CoV-2 RNA loads in Calu-3 cells infected with SARS-CoV-2 at a MOI of 0.5. All the data are presented as mean ± SEM and statistical significances are analyzed by one-way ANOVA. The results are representative two reproducible independent experiments, n = 3–4 in each group. Compared with WT, *P < 0.05; **P < 0.01; ***P < 0.001. MAVS mitochondrial antiviral signaling protein, MDA5 melanoma differentiation-associated protein 5, PFU plaque forming unit, RIG-I retinoic acid-inducible gene I. [file 40779_2021_340_MOESM2_ESM.tif]

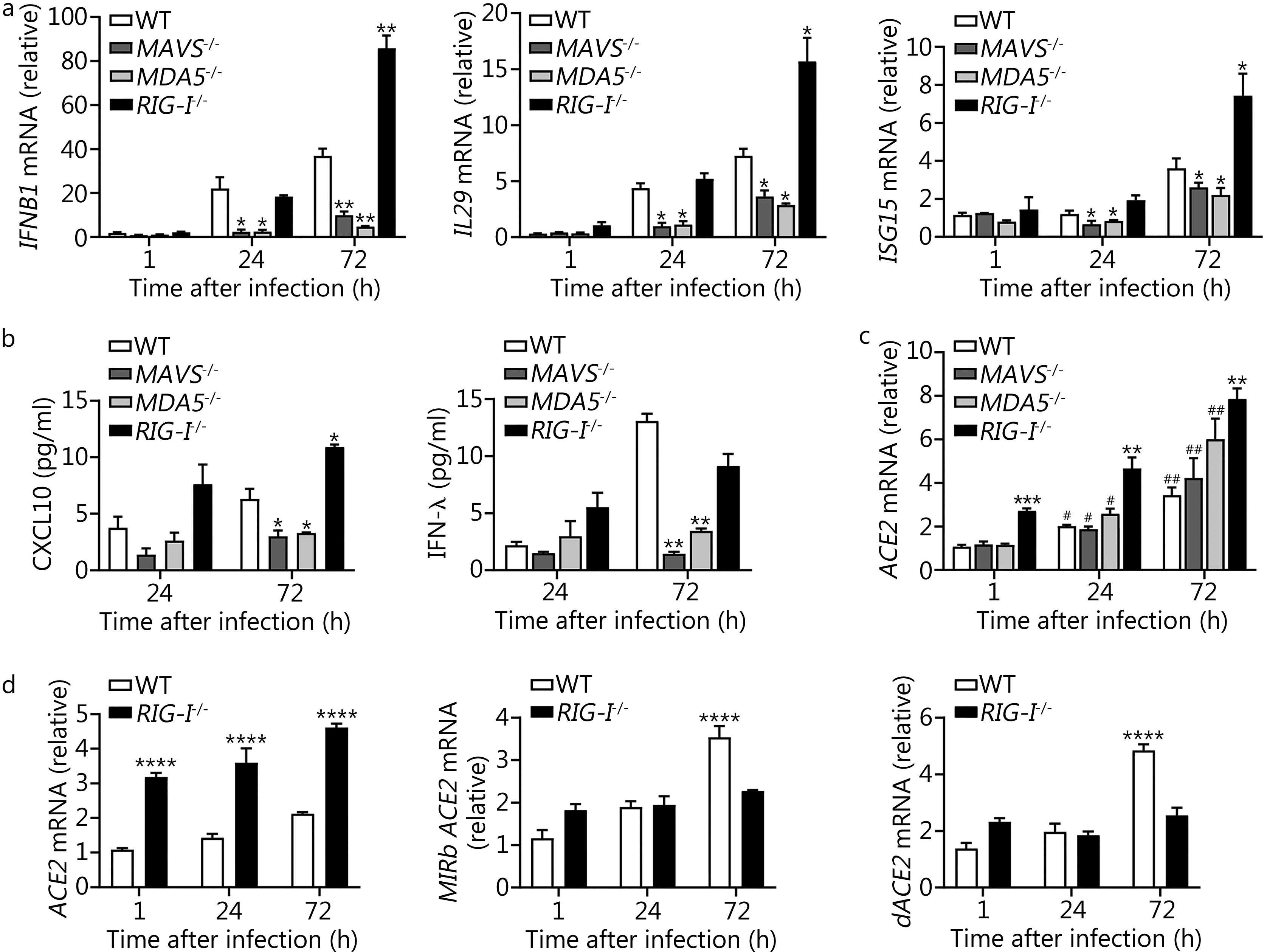

Supplement: Supplementary file 3 — Additional file 3: Fig. S3. An essential role of the MDA5-MAVS axis in induction of type I/III IFNs by SARS-CoV-2. a Quantitative RT-PCR analyses of immune gene transcripts. b Quantification of IFN-λ and CXCL10 proteins by ELISA, in Calu-3 cells infected with SARS-CoV-2 at a multiplicity of infection (MOI) of 0.5. c Quantitative RT-PCR analyses of full-length ACE2 mRNA. d The short isoform of ACE2. MIRb ACE2 and dACE2 are different designations for the same short isoform from two recent publications. All the data are presented as mean ± SEM and statistical significances are analyzed by one-way ANOVA (a and b), and non-parametric Mann–Whitney U test (d). The results are representative two reproducible independent experiments, n = 3 for each group. Compared with WT, *P < 0.05; **P < 0.01; ***P < 0.001; ****P < 0.0001. Compared with 1 h, #P < 0.05; ##P < 0.01. ACE2 angiotensin-converting enzyme 2, CXCL10 C-X-C motif chemokine ligand 10, IFNB1 type I IFN, IFN interferon, IL29 type III IFN; ISG15 interferon-stimulated gene 15, MAVS mitochondrial antiviral signaling protein, MDA5 melanoma differentiation-associated protein 5, RIG-I retinoic acid-inducible gene I. [file 40779_2021_340_MOESM3_ESM.tif]
